# Supplementary material for: Search of high-frequency variations of fundamental constants using spin-based quantum sensors
Source: Natl Sci Rev. 2025 Mar 8;12(4):nwaf085. doi: 10.1093/nsr/nwaf085 (PMC12012896; doi:10.1093/nsr/nwaf085)
Supplement: nwaf085_Supplemental_Files [file nwaf085_supplemental_files.zip › Supplementary Data.pdf]

Supplementary Data for
Search of High-Frequency variations of Fundamental Constants Using Spin Quantum Sensors

Contents

I. Supplementary Method 1: Theory to detect Fundamental Constant Variations by NV center 2
- A. Fundamental constants' time-varying caused by scalar field 2
- B. Effect of fundamental constant variation on NV center 2
- C. Principle to detect variations on energy levels by quantum mixing 3
- D. Modeling of the NV relaxation process 4

II. Supplementary Method 2: Experiment 5
- A. Setup 5
- B. Evaluation of the effectiveness of two photon process method 6
- C. Sample preparation and properties 8
- D. Experiment procedure 8
- E. Estimation for constrains of dark matter 10

References 11

The searches of fundamental constants plays a crucial role in enhancing our comprehension of the natural world. Previous studies has predominantly utilized atomic spectroscopy [S1–S4] and optomechanical systems [S5–S9] to search variations below 0.1 GHz. However, to investigate high-frequency variations in fundamental constants poses a significant experimental challenge, despite its crucial implications for various fundamental physics inquiries. Here we propose and implement an experiment harnessing spin quantum sensors to search high-frequency variations in fundamental constants, encompassing the fine structure constant and electron mass, in the previously uncharted frequency domain from 0.1 to 12 GHz. Our study yields constraints on their relative variations as low as 5 ppm and 8 ppm, respectively. Additionally, based on our results, we establish stringent upper limits on the coupling constants associated with scalar field dark matter from 0.4 μeV to 50 μeV. Our research underscores the potential for probing fundamental physics at high frequencies by spin quantum sensors.

I. SUPPLEMENTARY METHOD 1: THEORY TO DETECT FUNDAMENTAL CONSTANT VARIATIONS BY NV CENTER

A. Fundamental constants' time-varying caused by scalar field

The effect of time-varying fundamental constants is notable in the presence of a background scalar field. This scalar field can interact with the fields of standard model through various coupling, with common terms given by

$$\mathcal{L}_{\text{int}} \supset \frac{\phi}{\Lambda_\gamma}\frac{F_{\mu\nu}F^{\mu\nu}}{4} - \frac{\phi}{\Lambda_e} m_e \bar{\psi}_e \psi_e, \tag{S1}$$

where m_e is the electron rest mass, $F_{\mu\nu}$ is the electromagnetic field tensor, ψ_e and $\bar{\psi}_\text{e}$ are the electron field and its Dirac conjugate, and Λ_γ and Λ_e parameterize the coupling. The presence of a scalar field ϕ results in the oscillation of both α and m_e, a consequence of the field oscillating at the Compton frequency of the scalar field. When the timescales are shorter than the coherence time of the field, the field can be expressed as [S10, S11]

$$\phi(t) = \frac{\hbar\sqrt{2\hbar c \rho_{\text{DM}}}}{m_\phi c}\cos\left(\omega_\phi t\right), \tag{S2}$$

where $\rho_{\text{DM}} \approx 0.4$ GeV/cm^3, $\omega_\phi = \left(m_\phi c^2\right)/\hbar$ is the angular Compton frequency and m_ϕ is the mass of the field. The quantities α and m_e follow the oscillations of ϕ and can be written as

$$\alpha(t) = \alpha_0\left[1 + \frac{1}{\Lambda_\gamma}\phi(t)\right], \tag{S3}$$

$$m_e(t) = m_{e,0}\left(1 + \frac{1}{\Lambda_e}\phi(t)\right). \tag{S4}$$

B. Effect of fundamental constant variation on NV center

The nitrogen-vacancy (NV) center exhibits a spin-1 electron spin state described by the Hamiltonian

$$H = DS_z^2 + \gamma_e \mathbf{B}\cdot\mathbf{S} + A_\parallel S_z I_{z,\text{ N}} + A_\perp\left(S_x I_x + S_y I_y\right) + \mathcal{P}I_z^2 - \gamma_\text{N}\mathbf{B}\cdot\mathbf{I}, \tag{S5}$$

where the zero-field splitting $D/2\pi \approx 2.87$ GHz arises from the spin-spin interaction that perturbs the symmetry. Here, $\mathbf{B}$ represents the magnetic field vector, $\mathbf{S}$ denotes the NV electron spin operator, $\mathbf{I}$ stands for the adjacent nitrogen nuclear spin operator, $A_\parallel, A_\perp$ indicate the strengths of the parallel and perpendicular hyperfine couplings, $\mathcal{P}$ signifies the nuclear electric quadrupole coupling and γ_N is the gyromagnetic ratio of the respective nitrogen nuclear isotope.

An external magnetic field of B_0 =50 mT, generated by a permanent magnet, is applied to the system. This B_0 field induces the anti-crossing of the excited state levels of the NV center [S12], causing polarization of the nuclear spin to the $m_s = -1$ state. Subsequently, the system's Hamiltonian can be simplified, omitting the constant term, as,

$$H = DS_z^2 + (\gamma_e B_0 + A_\parallel)S_z + \gamma_e \mathbf{B}(t)\cdot\mathbf{S}. \tag{S6}$$

The NV system consists of three dangling bonds on the carbon atoms adjacent to the vacancy, along with two bonding electrons on the nitrogen atom and an additional electron trapped at the center [S13]. Consequently, the defect's electronic configuration can be described as two holes occupying double-generated orbitals, forming a spin-1 system. The ground-state splitting [S14, S15] of the NV center is

$$D \propto \frac{\mu_0 g_e^2 \mu_B^2}{4\pi} \left\langle \psi^{A_2} \middle| \frac{1-3z^2/r^2}{r^3} \middle| \psi^{A_2} \right\rangle, \tag{S7}$$

where μ_0 is the vacuum permeability, g_e is the g-value of NV electron spin, μ_B is Bohr magneton, $|\psi^{A_2}\rangle$ is the orbital part of the ground state $^3A_2$ wave function, r is the displacement between electrons and z is the projection of the distance vector along the principal axis. The effective displacement is $r_{\text{eff}} = a_0/\sqrt{2}$, where $a_0 = 3.56\,\text{Å}$ is the cubic lattice spacing of diamond, which is proportional to the Bohr radius $a_B = \hbar/(\alpha m_e c)$. As a consequence of the oscillating scalar dark matter field, the electron magnetic moment $\mu_B = e\hbar/(2m_e)$ undergoes oscillation as a result of the oscillating electron mass. The coupling between the spin magnetic moment μ_B yields a modification of the photon propagator [S16], resulting in the modification of the electromagnetic interaction between fermions which contains a product of $e^2 \propto \alpha$ and the photon propagator $1/\left[q^2\left(1-\Lambda_\gamma^{-1}\phi\right)\right]$, where q is the momentum transfer. Thus the ground-state splitting D yields

$$D \propto \alpha^4 m_e, \tag{S8}$$

which leads to,

$$\frac{\delta D}{D} = \frac{\delta m_e}{m_e} + \frac{4\delta\alpha}{\alpha}. \tag{S9}$$

In our experiment, the NV center is subjected to an external magnetic field created by a permanent magnet. Consequently, the energy level difference of the NV center is influenced not only by the zero-field splitting but also by the Zeeman effect of the external magnetic field B_0. As a result, the energy differences between the $|0\rangle$ state and the $|\pm 1\rangle$ states are denoted as $E_\pm/\hbar = D \pm \gamma_e B_0 = D \pm g_e \mu_B B_0/\hbar$. Considering the modification of the electromagnetic interaction between fermions, the variations in $\gamma_e B_0$ is thus

$$\frac{\delta\left(\gamma_e B_0\right)}{\gamma_e B_0} = -\frac{2\delta m_e}{m_e} + \frac{\delta\alpha}{\alpha}. \tag{S10}$$

In the condition where $\gamma_e B_0 \approx D/2$, the variation of $E_\pm$ is thus

$$\begin{aligned} \frac{\delta E_-}{E_-} &\approx 2\frac{\delta D}{D} - \frac{\delta(\gamma_e B_0)}{\gamma_e B_0} \\ &= \frac{7\delta\alpha}{\alpha} + \frac{4\delta m_e}{m_e}, \end{aligned} \tag{S11}$$

and

$$\begin{aligned} \frac{\delta E_+}{E_+} &\approx 2\frac{\delta D}{D} + \frac{\delta(\gamma_e B_0)}{\gamma_e B_0} \\ &= \frac{9\delta\alpha}{\alpha}. \end{aligned} \tag{S12}$$

Thus he high-frequency variation of the energy levels is influenced by both the variation δD and the variation of the Zeeman term $\delta(\gamma_e B_0)$. Therefore, the variation $\delta E_\pm$ is dependent on the variation $\delta\alpha$ of the fine structure constant and δm_e of the electron mass, making it sensitive for searching of their variations.

C. Principle to detect variations on energy levels by quantum mixing

As we search the variation of NV electron spin's energy levels, which corresponds to the magnetic field variation along the principal axis. Such energy level variation can be searched at low frequencies using techniques like dynamical decoupling and Hartmann-Hahn procedure. However, despite their theoretical feasibility, these methods require high microwave field power for high frequency signal, which may introduce technical problems like temperature

instability. For this experiment, we utilized a two-photon measurement technique to search the energy level variation $b_\phi \cos(\omega_\phi t) = \delta E_-/\hbar = \delta D - \delta(\gamma_e B_0)$.

In the experiment, the magnetic field is adjusted to 50 mT. The target signal frequency is ω_ϕ with effective field strength b_ϕ. Assisted by the bias a.c. field with frequency ω_1 and amplitude Ω_1, the system Hamiltonian is

$$H = DS_z^2 + (\gamma_e B_0 + A_\parallel)S_z + \sqrt{2}\Omega_1 S_x \cos(\omega_1 t) + \begin{pmatrix} \delta E_+/\hbar & 0 & 0 \\ 0 & 0 & 0 \\ 0 & 0 & b_\phi \cos(\omega_\phi t) \end{pmatrix}. \quad \text{(S13)}$$

Under specific conditions, the frequencies of the bias a.c. field and the scalar field can give rise to resonance, referred to as the phenomenon of two-photon resonance. To facilitate our discussion, we will focus on the queries related to the subspace created by the $m_s = 0$ and $m_s = -1$ states [S17]. The pseudo two level system Hamiltonian is thus

$$H = \frac{(D - \gamma_e B_0 - A_\parallel)}{2}\sigma_z + \Omega_1 \cos(\omega_1 t)\,\sigma_x + \frac{b_\phi \cos(\omega_\phi t)}{2}\sigma_z, \quad \text{(S14)}$$

where σ_j is Pauli matrices. Considering the rotating frame with frequency ω_1, the Hamitonian is

$$H' \approx \frac{\delta}{2}\sigma_z + \frac{\Omega_1}{2}\sigma_x + \frac{b_\phi \cos(\omega_\phi t)}{2}\sigma_z \quad \text{(S15)}$$

$$= H_z + H_{\text{MW}}, \quad \text{(S16)}$$

where $\delta = D - \gamma_e B_0 - A_\parallel - \omega_1 + \Omega_1^2/2\omega_1$, with the last term $\Omega_1^2/2\omega_1$ being Bloch-Sigert shift [S18, S19], $H_z = (\delta + b_\phi \cos(\omega_\phi t))\sigma_z/2$ is the σ_z component and $H_{\text{MW}} = \Omega_1\sigma_x/2$. The evolution arising from the diagonal components H_z can be found exactly, allowing us to move to the Floquet space, where the Hamiltonian becomes

$$\tilde{H}_{\text{MW}} = \begin{pmatrix} 0 & \sum_n A_n e^{-i(n\omega_\phi - \delta)t} \\ \sum_n A_n e^{i(n\omega_\phi - \delta)t} & 0 \end{pmatrix}, \quad \text{(S17)}$$

with $A_n = \Omega_1 J_n(b_\phi/\omega_\phi)/2$ [S20]. The resonant condition holds for $n = 1$ with $\Delta\omega = \omega_\phi - \delta = 0$, i.e.

$$\omega_1 = E_- - \omega_\phi + \Omega_1^2/2\omega_1. \quad \text{(S18)}$$

And under weak field approximation $b_\phi \ll \omega_\phi$

$$A_1 \approx \Omega_1 b_\phi/4\omega_\phi. \quad \text{(S19)}$$

Therefore, under the modulation of resonant bias a.c. field, the effect of the scalar field equivalent to the z-direction microwave field is transformed into a transverse driving field $\tilde{H}_{\text{MW}} \approx b_{\text{eff}}\sigma_x/2$, similar to Rabi oscillation, namely, the twin-photon process, with effective driving strength

$$b_{\text{eff}} = \frac{\Omega_1 b_\phi}{2\omega_\phi}. \quad \text{(S20)}$$

D. Modeling of the NV relaxation process

Under strong driving conditions, we can observe spin oscillation. However, for the measurement for searching weak signals, such as dark matter scalar field signals, it is imperative to consider the dephasing effects inherent to the spin itself. As a result, the environmental effects on NV must be taken into account. Consequently, the time evolution of the density matrix, $\rho_{\text{T}}(t)$, is described by

$$\dot{\rho}_{\text{T}}(t) = -i\left[H_{\text{T}}(t), \rho_{\text{T}}(t)\right] \quad \text{(S21)}$$

where $\rho_{\text{T}}(t)$ represents the combined density matrix of the entire NV spin, scalar field and environment system. The full Hamiltonian is given by $H_{\text{T}} = H + H_E$ where H_E is the environment Hamiltonian. The environment contribute a decoherence effect. As sensing processes occur on timescales that are much longer that the typical interaction timescales of the environmental constituents, essentially placing the system in a Markovian regime, the resulting decoherence will be purely exponential.

These effects may thus be modeled using a master equation approach for the reduced density matrix of the NV spin, ρ, as follows

$$\dot{\rho} = -i\left[H(t), \rho\right] + \left(\mathcal{L}\rho\mathcal{L}^{\dagger} - \frac{1}{2}\left\{\mathcal{L}^{\dagger}\mathcal{L}, \rho\right\}\right) \tag{S22}$$

where $\mathcal{L} = \sqrt{2\gamma_2}S_z$ is the Lindbladian operator corresponding to coherence process on NV spin. The decoherence rate is $\gamma_2 = 1/T_2$. After we change into Floquet basis, the equation is given by

$$\dot{\rho}_F(t) = -i\left[\tilde{H}_{\mathrm{MW}}(t), \rho_F(t)\right] + \gamma_2\left(S_z\rho_F(t)S_z - \rho_F(t)\right). \tag{S23}$$

According to Ref. [S21], the solution is

$$P_0\left(t\right) = \frac{1}{2} + \frac{1}{2}\exp\left(-\frac{\gamma_2 t}{2}\right)\left[\cosh\left(\frac{t}{4}\sqrt{\gamma_2^2 - 2b_{\mathrm{eff}}^2}\right) + \frac{\gamma_2}{\sqrt{\gamma_2^2 - 2b_{\mathrm{eff}}^2}}\sinh\left(\frac{t}{4}\sqrt{\gamma_2^2 - 2b_{\mathrm{eff}}^2}\right)\right] \tag{S24}$$

for resonant condition $\Delta\omega = 0$. In the weak interaction limit where $\gamma_2 \gg b_{\mathrm{eff}}$, we have

$$P_0(t)|_{\Delta\omega=0} = \frac{1}{2} + \frac{1}{2}\exp\left(-\frac{b_{\mathrm{eff}}^2}{2\gamma_2}t\right). \tag{S25}$$

and

$$P_0(t)|_{\Delta\omega=0} = \frac{1}{2} + \frac{1}{2}\exp\left(-\frac{b_{\mathrm{eff}}^2 t}{2}\frac{\gamma_2}{\gamma_2^2 + \Delta\omega^2}\right) \tag{S26}$$

for nonresonant condition. Hence the corresponding logitudinal relaxation rate is given by

$$\gamma_\phi = \frac{b_{\mathrm{eff}}^2}{2}\frac{\gamma_2}{\gamma_2^2 + \Delta\omega^2} \tag{S27}$$

II. SUPPLEMENTARY METHOD 2: EXPERIMENT

A. Setup

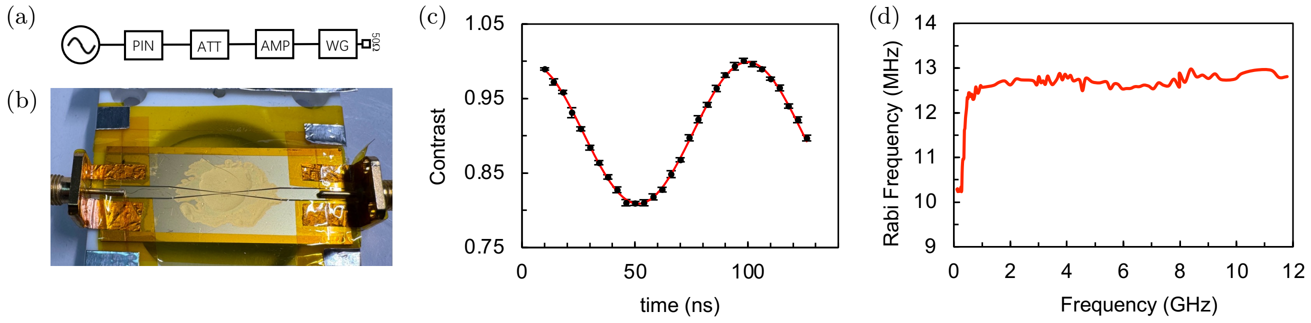

FIG. S1: (a) The schematic of the microwave setup shows the microwave emission from the source, controlled by a switch, amplifier, and then entering the coplanar waveguide. (b) Photo of the coplanar waveguide in correlation with the diamond sample. (c) Example of Rabi oscillation with resonant frequency of 110 MHz. (d) Illustration of Rabi oscillation frequencies at various resonance frequencies.

A home-built confocal optical system is utilized in our study. It employs a 532 nm laser (CNI, MLL-III-532-150mW) to excite the NV center and collect the resulting fluorescence through the same system. An APD (avalanche photodiode, SPCM-AQRH-14-FC) is employed for fluorescence detection. Additionally, our microwave system was designed to generate microwave pulses. These microwave pulses were then applied to the NV center to manipulate

its electron spin state. Initially, two microwave sources (R&S-SMB100B and Rigol DSG3000B) generated microwave signals of different frequencies. Following the control by a microwave switch (P1T-DC40G-65-T-292FF-1NS-OPT18G-50OHM) and a control sequence generator (Ciqtek, ASG-GT50-C), the microwave signals were combined using a power combiner. After amplification by a microwave amplifier (ZVA-183-S+), the combined signal was fed to the NV center on the diamond sample through a coplanar waveguide. The diamond sample was positioned on a three-dimensional nanopositioning stage (Physik Instrument), while the microscope objective (Olympus UPLXAPO100XO) remained fixed. The position of the sample was controlled using the nanopositioning stage. Additionally, a μm three-dimensional electric stage (Symc-tec, NS-XY200Z100-01) is used to position a permanent magnet above the diamond sample, enabling adjustment of the field strength and direction of the magnetic field on the NV center. Data acquisition is carried out using an National Instrument DAQ-6612 card.

As depicted in the Fig. S1(a), our microwave circuit begins at the microwave source, passes through a microwave switch, and is then amplified by an amplifier. After the amplifier, we feed the microwave onto the broadband coplanar waveguide (CPW). The CPW is illustrated in the Fig. S1(b). A 50 μm diamond sample is placed on top of the CPW. We adjust the position of the magnet using a micrometer stage to control the magnitude and direction of the magnetic field. By tuning the magnetic field towards the NV axis, as indicated by the resonant frequencies of $m_s = 0 \leftrightarrow m_s = -1$ and $m_s = 0 \leftrightarrow m_s = 1$ transitions as well as the fluorescence intensity, we calibrate the intensity of the bias a.c. field under broadband conditions. As shown in the Fig. S1(c), the microwave intensity remains consistent within a significantly broad bandwidth. The drop in microwave intensity on the left side occurs because it reaches the bandwidth limit of the amplifier at 0.7 GHz, although it continues to work.

B. Evaluation of the effectiveness of two photon process method

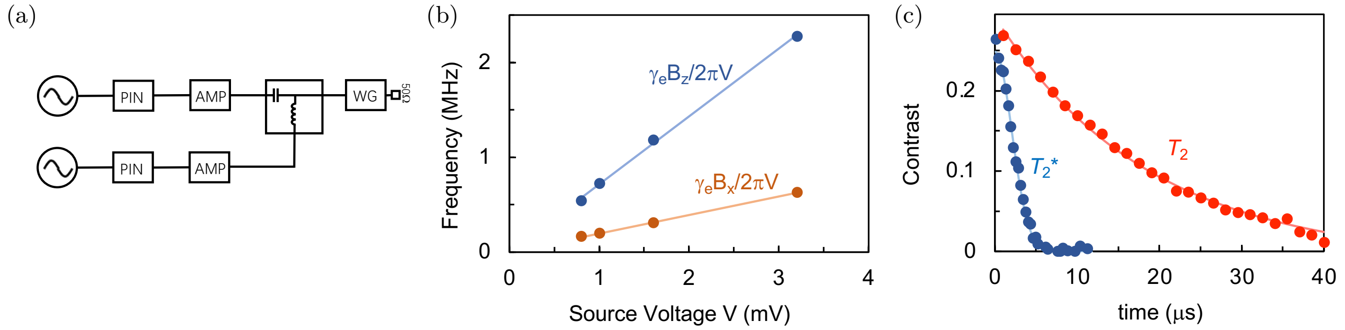

FIG. S2: (a) Microwave circuit diagram. Two different-frequency microwaves are combined through a diplexer into the waveguide. (b) Calibration of the x and z magnetic field responses relative to the source voltage using Rabi oscillations and dynamical decoupling. The response of the transverse microwave field are measured by Rabi frequency $\Omega_x/2\pi V_x = \gamma_e B_x/2\pi V_x = 0.196 \pm 0.04$ MHz/mV. The response of the longitude microwave field are measured by dynamical decoupling, with strength of $\Omega_z/2\pi V_z = \gamma_e B_z/2\pi V_z = 0.715 \pm 0.02$ MHz/mV. (c) Curves representing the free induction decay and T_2 of the new NV center spins. The blue curve is fitted by $f(t) = e^{-(t/T_2^*)^2}$, with $T_2^* = 3$ μs. The red curve is fitted by $g(t) = e^{-t/T_2}$, with $T_2 = 18$ μs.

To calibrate and evaluate the effectiveness of our method, we introduced a new microwave circuit and added an artificial field to simulate the scalar field effect in another NV center. In our experimental setup, we synthesized two different frequency microwave signals using a diplexer (Marki DPXN-1) and fed them into a broadband CPW for transmission to the NV center location (Fig. S2(a)). Two microwave amplifiers are used for the working frequency band. The strength of the transverse microwave field are measured by Rabi frequency $\Omega_x/2\pi V_x = \gamma_e B_x/2\pi V_x = 0.196 \pm 0.04$ MHz/mV versus the microwave source voltage (Fig. S2(b)). The strength of the longitude microwave field are measured by standard quantum sensing protocol, dynamical decoupling, $\Omega_z/2\pi V_z = \gamma_e B_z/2\pi V_z = 0.715 \pm 0.02$ MHz/mV versus the microwave source voltage (Fig. S2(b)). The decoherence time $T_2 = 18$ μs and free-induced decay time $T_2^* = 3$ μs induced by line-width are measured for this new NV center (Fig. S2(c)).

Here we use two microwave fields with different frequencies to realized two photon process. During the experiment, we initially set the external static magnetic field to 50 mT to polarize the nuclear spin state and achieve a resonance frequency of 1.42 GHz for the NV center electron spin. Subsequently, we applied an artificial microwave field in the z-direction with a frequency of $\omega_\phi/2\pi = 100$ MHz and a bias a.c. field with a frequency of approximately 1.32 GHz, equivalent to $\Omega_1/2\pi = 10$ MHz in strength. The combination of these two microwave fields aims to manipulate the electron spin state of the NV center. Based on our theoretical model and calculations (Sec. I C), under resonance conditions where the frequency of the bias a.c. field matches that of the artificial microwave field, we can excite

oscillations of the population between $m_s = 0$ and $m_s = -1$ through a two-photon process (Fig. S3(a)). According to Eq. S20,

$$\frac{b_{\rm eff}}{b_\phi} = \frac{\Omega_1}{2\omega_\phi} = 0.05 \tag{S28}$$

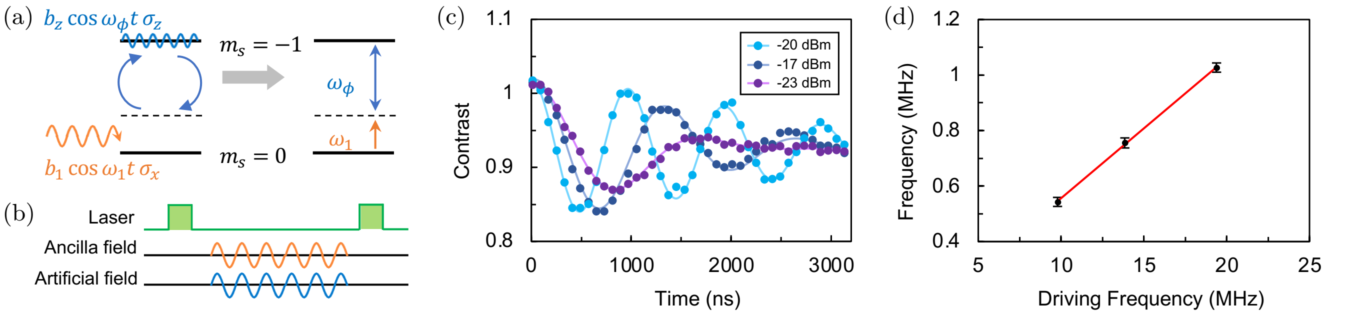

FIG. S3: (a) On the left, an artificial field induces energy level perturbations along the NV principal axis. These perturbations are measured by applying a bias a.c. field in the perpendicular direction. On the right, under resonance conditions, energy level perturbations along the principal axis (z-axis) and the bias a.c. field in the perpendicular direction are transformed into transitions between two energy levels. (b) The pulse control sequence was utilized. (c) By simultaneously applying artificial and bias a.c. field, oscillatory curves were observed. Changes in the strength of the artificial field led to the measurement of various curves. (d) The relationship between oscillation frequency and artificial field intensity was determined, obtaining a slope of 0.0503 ± 0.009 through linear fit.

Corresponding oscillations were observed in the experiment, as depicted in Fig. S3(c). By varying the strength of the applied field, a decrease in oscillation frequency was noted with decreasing artificial field strength. The relationship between the artificial field strength b_ϕ and the effective strength $b_{\rm eff}$ was plotted in Fig. S3(d), showing a clear proportional relationship between oscillation frequency and artificial field strength as illustrated above, with a slope of 0.0503 ± 0.009, consistent with the theoretical expectations.

The previously described scenario exemplifies a situation characterized by strong field strength. In practical experiments, where we are measuring a small signal, it necessitates an evaluation of responses under limited quantities. The applied magnetic field strength was decreased, leading to the effective field strength $b_{\rm eff}$ corresponding to being less than $1/T_2$. This adjustment led to the observation of the shift from coherent oscillations to decay, as illustrated in the Fig. S4(a). The coherent-driving effect leads faster decay remains observable at evolution time $t < T_2$ in stronger fields, as depicted graphically. Hence, we applied a double exponential decay fitting to the decay curves of the three strongest fields and single exponential fitting to the others. We obtained the relationship between the decay rate and the applied field strength for evolution time $t > T_2$, as illustrated in the Fig. S4(b). The relationship is quadratic, meet with Eq. S27, with a coefficient of $\beta = (1.16 \pm 0.13) \times 10^{-3}$ kHz^{-1}. Considering the decoherence of NV center with rate $\gamma_2 = 1/T_2$, and the spectral line broadening $\Gamma_2 = 2/T_2^*$, we computed the decay behavior according to Eq. S27,

$$\gamma_\phi = \frac{1}{\sqrt{\pi}\Delta\omega} \int_{-\infty}^{+\infty} \frac{b_{\rm eff}^2 \gamma_2}{2\left(\gamma_2^2 + \omega^2\right)} e^{-\omega^2/\Gamma_2^2} d\omega. \tag{S29}$$

The quadratic coefficient is calculate as $\beta = 1.19 \times 10^{-3}$ kHz^{-1}, which meets well with the experimental data. We focus not only on the resonant behavior of spin system under resonant conditions but also investigate the dynamics of the systems when deviating from the resonance frequency. The off-resonance decay rate is thus

$$\gamma_\phi = \frac{1}{\sqrt{\pi}\Delta\omega} \int_{-\infty}^{+\infty} \frac{b_{\rm eff}^2 \gamma_2}{2\left(\gamma_2^2 + (\omega - \Delta\omega)^2\right)} e^{-\omega^2/\Gamma_2^2} d\omega, \tag{S30}$$

where $\Delta\omega$ is the detuning frequency. Considering $\gamma_2 \ll \Gamma_2$, the Lorentzian line width is much smaller than the frequency range of interest, we can approximate it as a Dirac delta function. The off-resonance decay rate is thus approximately

$$\gamma_\phi \approx \frac{\sqrt{\pi}\Omega_1^2 b_\phi^2}{2\omega_\phi^2 \Gamma_2} e^{-\Delta\omega^2/\Gamma_2^2}, \tag{S31}$$

with linewidth $\Gamma_2 = 2\pi \cdot 106$ kHz, meet with 96 ± 6 kHz from the fitting result in Fig. S4(c).

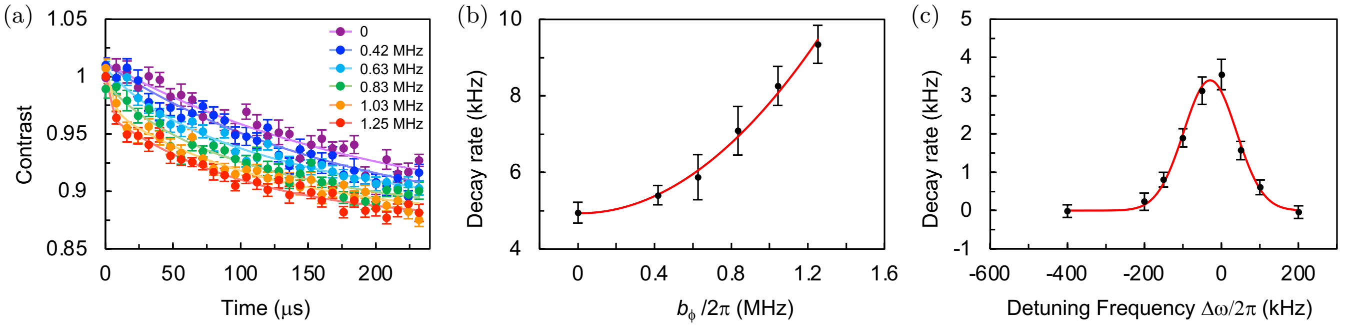

FIG. S4: (a) We decreased the artificial field b_ϕ significantly for measurements, observing a transition of the spin response from oscillation to decay. The curves in red, orange, and green were fitted with a double exponential function, while the others were fitted with a single exponential function. (b) Relationship between the decay rate and the artificial field strength b_ϕ. (c) Relationship between the decay rate and off-resonance detuning frequency $\Delta\omega/2\pi$. This curve has been baseline-subtracted.

C. Sample preparation and properties

The NV sensor utilized in our experiment is a single-crystal diamond film, cut into 50 μm pieces and annealed for two hours at 800 °C. The implantation energy is set at approximately 50 keV, resulting in single NV centers located roughly 62 nm from the surface. The implantation dosage is $10^9/\text{cm}^2$, corresponding to $10-15$ individual NV centers within a $10{\times}10$ μm^2 area. The NV center employed for our dark matter experiment is depicted in the Fig. S5(a). The brighter intensity of this particular oriented NV center is attributed to the excited-state level anti-crossing [S12] induced by a 50 mT external magnetic field. The FID time (Ramsey fringe) and T_1 time of the NV center are measured shown in the Fig. S5(b) and (c).

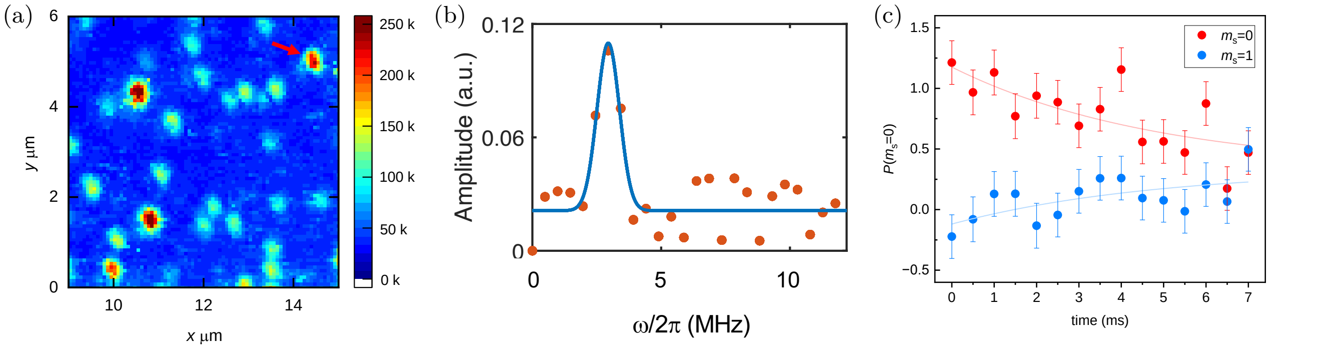

FIG. S5: (a) Fluorescence density map. NV centers were observed using confocal microscopy, with the arrow indicating the NV sensor employed in our experiment. Color bar indicates the number of photons collected per second. (b) Fourier transformation of the Ramsey fringe experiment that determines the linewidth $\Gamma_2/2\pi = 0.62$ MHz of the spin quantum sensor, fitted with Gaussian shape $f(\omega) = e^{-(\omega-\omega_c)^2/\Gamma_2^2}$. (c) Measurement of the original depolarization of the NV center with T_1 =4.8 ms.

D. Experiment procedure

The high-frequency energy level variation $\delta E_-/\hbar = b_\phi \cos(\omega_\phi t)$, with effective variation amplitude b_ϕ, is detected through a quantum mixing process (Fig. S6(b)). The NV center spin states are driven into virtual levels in Floquet space by a bias a.c. field with an amplitude of Ω_1 and frequency of ω_1, which is applied perpendicular to the NV's principle axis. These new virtual levels become resonant with the target signal field $b_\phi \cos(\omega_\phi t)$ under the condition

of

$$\omega_1 = E_- - \omega_\phi + \Omega_1^2/2\omega_1. \tag{S32}$$

The frequency of the target signal field, ω_ϕ, is swept from 0.1 GHz to 12 GHz by adjusting the frequency of the bias a.c. field to meet resonance condition. The effective strength [S17] of the target field on the spin quantum sensor can be obtained as Eq. S20

$$b_{\text{eff}} = \Omega_1 b_\phi / 2\omega_\phi. \tag{S33}$$

Taking into account the spectral broadening of the spin quantum sensor $\Gamma_2 = 2/T_2^*$, the energy level variation causes the electron spin to depolarize at an additional rate of $\gamma_\phi = \sqrt{\pi}(\Omega_1 b_\phi)^2/2\omega_\phi^2\Gamma_2$ (Fig. S6(c)), given that the variations b_ϕ is always satisfied in weak signal detection scenarios. Therefore, by measuring experimentally the additional decay rate γ_ϕ of the NV center spin due to δE_-, the amplitude of the energy level variations can be obtained, allowing the estimation of variations in fundamental constants α, m_e at specific frequencies, as

$$b_\phi = \frac{\sqrt{2}\omega_\phi}{\sqrt[4]{\pi}\Omega_1}\sqrt{\gamma_\phi \Gamma_2}. \tag{S34}$$

The background depolarization of the NV center spin due to spin-lattice relaxation is removed by comparing the relaxation rate measured at frequency with differences greater than 10 MHz, serving as a reference baseline for deduction. Finally, from the difference in relaxation rates between resonant and non-resonant frequencies, Through adjusting the frequency of the bias a.c. field, we can estimate the upper limit of potential variations in fundamental constants (see Sec. II.E for details) from 0.1 GHz to 12 GHz. We plot the upper limit γ_ϕ^{up} of the relaxation rate γ_ϕ in Fig. 1(D) of main text with a 95% confidence level (equivalent to a 2σ standard deviation). We note that our experiment covered the frequency range of $\omega_\phi/2\pi$ from 0.1 GHz to 12 GHz, comprising a experiments for 147 different ω_ϕ. The bias a.c. field frequency, $\omega_1/2\pi$, ranged from approximately 0.42 GHz to 10.58 GHz according to Eq. S1, with field strength $\Omega_1/2\pi$ about 10 MHz (refer to Fig. S1). Each point took about eight hours to build up the statistics, and the whole experiment took about one month.

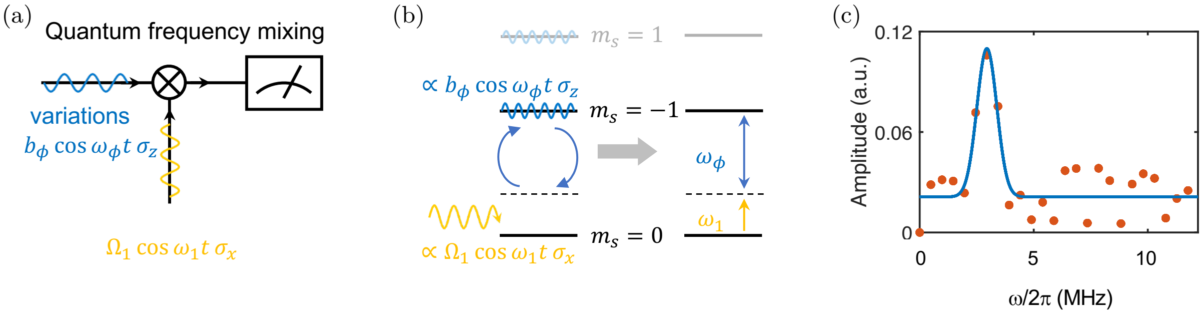

FIG. S6: (a) Schematic diagram of the experiment. The NV center serves as a quantum mixer to mix the variation signal (blue) with the bias a.c. field (orange) [S17]. (b) On the left, variations of fundamental constants cause energy level perturbations along the NV principle axis. The energy perturbations are measured by applying a bias a.c. field in the perpendicular direction. On the right, the energy level perturbations along the principle axis (z-axis) and the bias a.c. field in the perpendicular direction, under resonance conditions, are transformed into transitions between two levels. In the condition of finite spectral broadening, these transitions are equivalent to the additional spin longitudinal relaxation. (c) Fourier transformation of the Ramsey fringe experiment that determines the linewidth $\Gamma_2/2\pi = 0.62$ MHz of the spin quantum sensor, fitted with Gaussian shape $f(\omega) = e^{-(\omega-\omega_c)^2/\Gamma_2^2}$.

By utilizing the additional relaxation rate γ_ϕ, along with the equations (S11) and (S34), and considering the frequency response characteristics of the NV spin, we establish a relationship between the spin relaxation rate and the fundamental constants. The assessments of the relaxation rate γ_ϕ allow us to estimate the upper limits on magnitudes of high-frequency variations in these constants. The high-frequency variations are then constrained within a 95% confidence level, equivalent to a 2σ standard deviation. The experimental results are depicted in Fig. 1(E) of main text, where the jagged features in the curve are due to the near-resonant response of spin quantum sensor with scaling $\sim \exp(\Delta\omega^2/2\Gamma_2^2)$ (see Sec. II.B for details). The results demonstrate that within the frequency range of 0.1 GHz to 12 GHz, the best constraint on variations in the fundamental constants is restricted to approximately 10^{-6} to 10^{-3} in α and m_e.

E. Estimation for constrains of dark matter

The linewidth of the scalar field is limited, typically $\sim 10^{-6}\omega_\phi$ for dark matter, and the linewidth of the spin system is 1 MHz. Thus, we measure the reference decay rate, $\gamma_{\rm ref}$, at a frequency of $\omega_{\rm ref}/2\pi = \omega_\phi/2\pi - 10$ MHz. This frequency difference is at least one order larger than the linewidth. Consequently, the nonresonant decay rate is two orders smaller than the resonant decay rate (Eq. S31). Thus the scalar field induced spin state decay is obtained as

$$\Gamma_\phi(t) = \frac{P_0(t) - P_{-1}(t)}{P_0^{\rm ref}(t) - P_{-1}^{\rm ref}(t)} = e^{-(\gamma_\phi - \gamma_{\rm ref})t} \approx e^{-\gamma_\phi t}, \tag{S35}$$

where $P_0^{\rm ref}(t)$ and $P_{-1}^{\rm ref}(t)$ is the spin state decay on reference frequency ω_ϕ, and decay rates induced by the scalar field are estimated from $\gamma_\phi^{\rm exp} = -\ln\Gamma_\phi/t$. The measurement time is set to 3 ms according to the intrinsic depolarization rate.

With an NV center count rate of 100 kcts, each experiment produces 0.03 photons during a single readout time of 300 ns. The single experiment run is repeated 3.2 million times to build statistics. We calculated the spin depolarization decay rate caused by the scalar field, based on experimentally obtained data and Eq. S35, and determined a 95% confidence interval. If γ_ϕ is less than 0, we regard it as a non-physical result and assign it a value of 0. We then estimate the upper limit of γ_ϕ by using

$$\gamma_\phi^{\rm up} = \max\{\gamma_\phi^{\rm exp}, 0\} + 2\sigma\left(\gamma_\phi^{\rm exp}\right). \tag{S36}$$

Utilizing the depolarization rate and Eq. 3, we obtain the upper limit of energy level fluctuations as follows:

$$b_\phi < \frac{\sqrt{2}\omega_\phi}{\sqrt[4]{\pi}\Omega_1}\sqrt{\gamma_\phi^{\rm up}\Gamma_2 e^{-\Delta\omega^2/\Gamma_2^2}} \tag{S37}$$

According to Eq. 3 and Eq. S11, the variation of E_- is

$$\frac{\delta E_-}{\hbar} = \frac{E_-}{\hbar}\left(\frac{7\delta\alpha}{\alpha} + \frac{4\delta m_e}{m_e}\right) < \frac{\sqrt{2}\omega_\phi}{\sqrt[4]{\pi}\Omega_1}\sqrt{\gamma_\phi^{\rm up}\Gamma_2 e^{-\Delta\omega^2/\Gamma_2^2}}, \tag{S38}$$

which set the upper limits of the fundamental constant variations,

$$\frac{\delta\alpha}{\alpha} < \frac{\sqrt{2}\omega_\phi}{7\sqrt[4]{\pi}E_-\Omega_1}\sqrt{\gamma_\phi^{\rm up}\Gamma_2 e^{-\Delta\omega^2/\Gamma_2^2}} \tag{S39}$$

and

$$\frac{\delta m_e}{m_e} < \frac{\sqrt{2}\omega_\phi}{4\sqrt[4]{\pi}E_-\Omega_1}\sqrt{\gamma_\phi^{\rm up}\Gamma_2 e^{-\Delta\omega^2/\Gamma_2^2}}. \tag{S40}$$

And in combination with Eq. S2,

$$\Lambda_\gamma^{-1} < \frac{\sqrt{2}\omega_\phi^2}{7\sqrt[4]{\pi}E_-\Omega_1 c}\sqrt{\frac{\gamma_\phi^{\rm up}\Gamma_2 e^{-\Delta\omega^2/\Gamma_2^2}}{2\hbar c\rho_{\rm DM}}} \tag{S41}$$

$$\Lambda_e^{-1} < \frac{\sqrt{2}\omega_\phi^2}{4\sqrt[4]{\pi}E_-\Omega_1 c}\sqrt{\frac{\gamma_\phi^{\rm up}\Gamma_2 e^{-\Delta\omega^2/\Gamma_2^2}}{2\hbar c\rho_{\rm DM}}} \tag{S42}$$

The tests of the relaxation rate γ_ϕ allow us to estimate the upper limits on magnitudes of high-frequency variations in these constants. The high-frequency variations are then bounded within a 95% confidence level, equivalent to a 2σ standard deviation.

Furthermore, we analyzed the scaling relationship between the measured $\delta\alpha/\alpha$ and $\delta m_e/m_e$ and the number of experimental runs, denoted as N, as depicted in Fig. S8. In our experiment, the predominant source of statistical fluctuations arises from photon shot noise, where $\sigma(I)/I \propto 1/\sqrt{N}$, with I representing the collected photon number and N indicating the experimental runs. In the scenario of a sufficiently small signal, the Eq. S35 simplifies to $\Gamma_\phi(t) \approx 1 - \gamma_\phi^{\rm exp}t$. Here, $\Gamma_\phi(t)$ symbolizes the normalized fluorescent contrast, leading to $\sigma(\gamma_\phi^{\rm exp}) \sim \sigma(\Gamma_\phi(t))/t \propto \sigma(I)/I \propto 1/\sqrt{N}$. Consequently, our estimations of the smallest detectable values for $\delta\alpha/\alpha$ and $\delta m_e/m_e$, which scale as $\sqrt{\gamma_\phi}$, can be represented as

$$\left(\frac{\delta\alpha}{\alpha}\right)_{\rm min}, \left(\frac{\delta m_e}{m_e}\right)_{\rm min} \propto \frac{1}{\sqrt[4]{N}}, \tag{S43}$$

which meet well with our experiment.

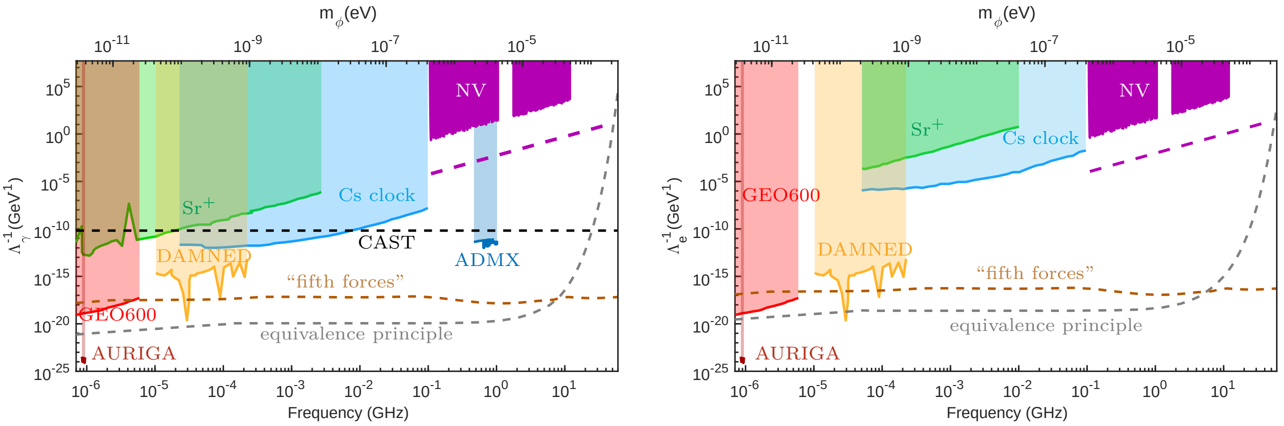

FIG. S7: **Constraints on the coupling parameters Λ_γ^{-1} and Λ_e^{-1} as a function of the mass of the field m_ϕ and Compton frequency for scalar field dark matter.** The left plot displays the constraints on the photon coupling Λ_γ, while the right plot illustrates the constraints on the electron coupling Λ_e, both established at a 95% confidence level. The legends in both plots maintain consistency. The violet region indicates the parameter space excluded in this experiment. The dashed violet line corresponds to the simulated results from ensemble spin sensor. Other colored regions indicate parameter spaces excluded in previous experiments: GEO600 [S9] (red), AURIGA [S8] (dark red), DAMNED [S22] (orange), dynamical decoupling in Sr^+ optical clock [S23] (green), Cs clock in cavity [S24] (blue), and ADMX [S25] (navy). The black dashed line corresponds to the results obtained from the CAST search for axions [S26], while the grey dashed line represents constraints derived from tests of the equivalence principle [S27, S28]. The brown dashed line corresponds to constraints resulting from searches for "fifth forces" [S29–S31]. Refer to Supplementary Table 2 for values.

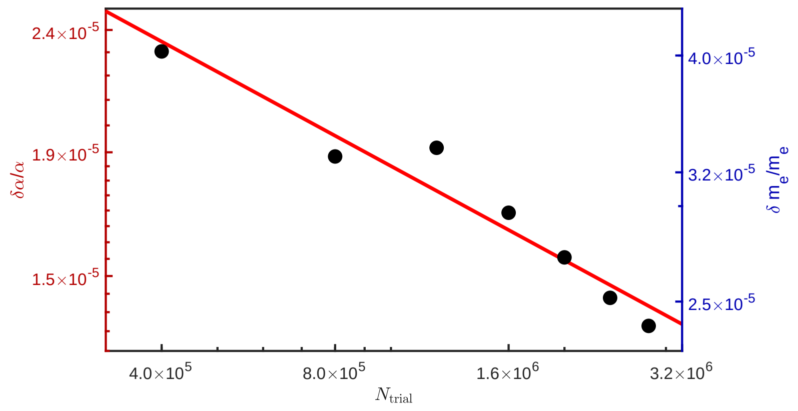

FIG. S8: Allan deviation for an experiment with $\omega_\phi/2\pi = 220$ MHz. The slope of the red line is fitted with -0.27 ± 0.04, aligning well with the theoretical prediction for the relationship between the smallest detectable signal and the number of experimental trials $N_{\text{trail}}^{-1/4}$.

[S1] A. Arvanitaki, J. Huang, and K. Van Tilburg, Phys. Rev. D **91**, 015015 (2015).
[S2] K. Van Tilburg, N. Leefer, L. Bougas, and D. Budker, Phys. Rev. Lett. **115**, 011802 (2015).
[S3] A. Hees, J. Guéna, M. Abgrall, S. Bize, and P. Wolf, Phys. Rev. Lett. **117**, 061301 (2016).
[S4] X. Zhang, A. Banerjee, M. Leyser, G. Perez, S. Schiller, D. Budker, and D. Antypas, Phys. Rev. Lett. **130**, 251002 (2023).
[S5] Y. V. Stadnik and V. V. Flambaum, Phys. Rev. Lett. **114**, 161301 (2015).
[S6] Y. V. Stadnik and V. V. Flambaum, Phys. Rev. A **93**, 063630 (2016).
[S7] W. M. Campbell, B. T. McAllister, M. Goryachev, E. N. Ivanov, and M. E. Tobar, Phys. Rev. Lett. **126**, 071301 (2021).
[S8] A. Branca, M. Bonaldi, M. Cerdonio, L. Conti, P. Falferi, F. Marin, R. Mezzena, A. Ortolan, G. A. Prodi, L. Taffarello, et al., Phys. Rev. Lett. **118**, 021302 (2017).
[S9] S. M. Vermeulen, P. Relton, H. Grote, V. Raymond, C. Affeldt, F. Bergamin, A. Bisht, M. Brinkmann, K. Danzmann, S. Doravari, et al., Nature **600**, 424 (2021).
[S10] A. Banerjee, H. Kim, and G. Perez, Phys. Rev. D **100**, 115026 (2019).

[S11] D. Antypas, O. Tretiak, A. Garcon, R. Ozeri, G. Perez, and D. Budker, Phys. Rev. Lett. **123**, 141102 (2019).
[S12] M. Steiner, P. Neumann, J. Beck, F. Jelezko, and J. Wrachtrup, Phys. Rev. B **81**, 035205 (2010).
[S13] Á. Gali, Nanophotonics **8**, 1907 (2019).
[S14] A. Lenef and S. C. Rand, Phys. Rev. B **53**, 13441 (1996).
[S15] M. W. Doherty, N. B. Manson, P. Delaney, and L. C. L. Hollenberg, New J. Phys. **13**, 025019 (2011).
[S16] I. M. Bloch, D. Budker, V. V. Flambaum, I. B. Samsonov, A. O. Sushkov, and O. Tretiak, Phys. Rev. D **107**, 075033 (2023).
[S17] G. Wang, Y.-X. Liu, J. M. Schloss, S. T. Alsid, D. A. Braje, and P. Cappellaro, Phys. Rev. X **12**, 021061 (2022).
[S18] F. Bloch and A. Siegert, Phys. Rev. **57**, 522 (1940).
[S19] J. Zhang, S. Saha, and D. Suter, Phys. Rev. A **98**, 052354 (2018).
[S20] S. Ashhab, J. R. Johansson, A. M. Zagoskin, and F. Nori, Phys. Rev. A **75**, 063414 (2007).
[S21] L. T. Hall, P. Kehayias, D. A. Simpson, A. Jarmola, A. Stacey, D. Budker, and L. C. L. Hollenberg, **7**, 10211 (2016).
[S22] E. Savalle, A. Hees, F. Frank, E. Cantin, P.-E. Pottie, B. M. Roberts, L. Cros, B. T. McAllister, and P. Wolf, Phys. Rev. Lett. **126**, 051301 (2021).
[S23] S. Aharony, N. Akerman, R. Ozeri, G. Perez, I. Savoray, and R. Shaniv, Phys. Rev. D **103**, 075017 (2021).
[S24] D. Antypas, O. Tretiak, A. Garcon, R. Ozeri, G. Perez, and D. Budker, Phys. Rev. Lett. **123**, 141102 (2019).
[S25] V. V. Flambaum, B. T. McAllister, I. B. Samsonov, and M. E. Tobar, Phys. Rev. D **106**, 055037 (2022).
[S26] V. Anastassopoulos, S. Aune, K. Barth, A. Belov, H. Bräuninger, G. Cantatore, J. M. Carmona, J. F. Castel, S. A. Cetin, F. Christensen, et al., Nature Phys **13**, 584 (2017).
[S27] J. Bergé, P. Brax, G. Métris, M. Pernot-Borràs, P. Touboul, and J.-P. Uzan, Phys. Rev. Lett. **120**, 141101 (2018).
[S28] A. Hees, O. Minazzoli, E. Savalle, Y. V. Stadnik, and P. Wolf, Phys. Rev. D **98**, 064051 (2018).
[S29] E. G. Adelberger, B. R. Heckel, and A. E. Nelson, Annu. Rev. Nucl. Part. Sci. **53**, 77 (2003).
[S30] E. Fischbach and C. Talmadge, arXiv:hep-ph/9606249 (1996).
[S31] A. S. Konopliv, S. W. Asmar, W. M. Folkner, Ö. Karatekin, D. C. Nunes, S. E. Smrekar, C. F. Yoder, and M. T. Zuber, Icarus **211**, 401 (2011).
